# Superior anti-tumor activity of the MDM2 antagonist idasanutlin and the Bcl-2 inhibitor venetoclax in p53 wild-type acute myeloid leukemia models

**DOI:** 10.1186/s13045-016-0280-3

**Published:** 2016-06-28

**Authors:** Christian Lehmann, Thomas Friess, Fabian Birzele, Anna Kiialainen, Markus Dangl

**Affiliations:** Roche Pharma Research & Early Development, Roche Innovation Center Munich, Roche Diagnostics GmbH, Nonnenwald 2, 82377 Penzberg, Germany; Roche Pharma Research & Early Development, Roche Innovation Center Basel, F-Hoffmann-La Roche Ltd, Basel, Switzerland; Present address: Medigene Immunotherapies GmbH, Planegg, Martinsried Germany

**Keywords:** BCL-2, MDM2, AML, Cell cycle kinetics, Apoptosis, Idasanutlin, Venetoclax, p53, MCL-1, Synergism

## Abstract

**Background:**

Venetoclax, a small molecule BH3 mimetic which inhibits the anti-apoptotic protein Bcl-2, and idasanutlin, a selective MDM2 antagonist, have both shown activity as single-agent treatments in pre-clinical and clinical studies in acute myeloid leukemia (AML). In this study, we deliver the rationale and molecular basis for the combination of idasanutlin and venetoclax for treatment of p53 wild-type AML.

**Methods:**

The effect of idasanutlin and venetoclax combination on cell viability, apoptosis, and cell cycle progression was investigated in vitro using established AML cell lines. In vivo efficacy was demonstrated in subcutaneous and orthotopic xenograft models generated in female nude or non-obese diabetic/severe combined immunodeficiency (NOD/SCID) mice. Mode-of-action analyses were performed by means of cell cycle kinetic studies, RNA sequencing as well as western blotting experiments.

**Results:**

Combination treatment with venetoclax and idasanutlin results in synergistic anti-tumor activity compared with the respective single-agent treatments in vitro, in p53 wild-type AML cell lines, and leads to strongly superior efficacy in vivo, in subcutaneous and orthotopic AML models. The inhibitory effects of idasanutlin were cell-cycle dependent, with cells arresting in G1 in consecutive cycles and the induction of apoptosis only evident after cells had gone through at least two cell cycles. Combination treatment with venetoclax removed this dependency, resulting in an acceleration of cell death kinetics. As expected, gene expression studies using RNA sequencing showed significant alterations to pathways associated with p53 signaling and cell cycle arrest (CCND1 pathway) in response to idasanutlin treatment. Only few gene expression changes were observed for venetoclax treatment and combination treatment, indicating that their effects are mediated mainly at the post-transcriptional level. Protein expression studies demonstrated that inhibition of the anti-apoptotic protein Mcl-1 contributed to the activity of venetoclax and idasanutlin, with earlier inhibition of Mcl-1 in response to combination treatment contributing to the superior combined activity. The role of Mcl-1 was confirmed by small hairpin RNA gene knockdown studies.

**Conclusions:**

Our findings provide functional and molecular insight on the superior anti-tumor activity of combined idasanutlin and venetoclax treatment in AML and support its further exploration in clinical studies.

**Electronic supplementary material:**

The online version of this article (doi:10.1186/s13045-016-0280-3) contains supplementary material, which is available to authorized users.

## Background

Acute myeloid leukemia (AML) is the most common acute leukemia in adults and, although high remission rates are achieved following induction chemotherapy, the majority of patients eventually relapses and becomes resistant to treatment [1]. There have been few changes in standard induction therapy and response rate over the past three decades, particularly for older patients who are ineligible for intensive chemotherapy or allogeneic stem cell transplantation [2–4]. Thus, the development of novel therapeutics based on an improved understanding of the molecular pathogenesis of AML has been a major focus to address this unmet need.

Idasanutlin (RG7388) and venetoclax (ABT-199/GDC-0199) have each shown significant activity in AML models in pre-clinical studies [5, 6], and each compound is currently under evaluation in patients with AML in phase I and II clinical trials as monotherapy or in combination with chemotherapy (study identifiers: NCT01773408; NCT01994837; NCT02203773; NCT02287233). In addition, venetoclax has recently been granted breakthrough designation by United States Food and Drug Administration (FDA) for treatment of relapsed and refractory chronic lymphoid leukemia (CLL) with 17p deletion [7].

Idasanutlin and venetoclax have complementary mechanisms of action. Idasanutlin is a second-generation, nutlin-class, orally bioavailable, selective mouse double minute 2 homolog (MDM2) antagonist [8]. MDM2 is an important negative regulator of the p53 tumor suppressor [9] and is expressed at high levels in a large proportion of AML [10, 11]. Wild-type p53 is expressed in over 80 % of AML cases; thus, inhibition of the interaction between MDM2 and p53 can re-establish the p53 pathway in AML cells resulting in cell cycle arrest and induction of apoptosis [10, 12, 13]. Venetoclax (GDC-0199/ABT-199) is an orally bioavailable novel small-molecule BCL-2 homolog domain 3 (BH3) mimetic, designed to selectively inhibit the anti-apoptotic protein Bcl-2. High Bcl-2 expression in AML is associated with resistance to chemotherapy and reduced likelihood of achieving complete remission [13–16]. Pre-clinical studies show that venetoclax has significant anti-tumor activity in AML models and that venetoclax-dependent inhibition of Bcl-2 can prime AML cells for responsiveness to chemotherapy [17].

The re-establishment of complementary key tumor suppressor and apoptotic pathways through combination treatment with venetoclax and idasanutlin may increase anti-tumor activity in AML cells. Direct activation of mitochondrial apoptosis through Bcl-2 inhibition may be enhanced by p53-dependent cell cycle arrest, p53-induced transcription of the pro-apoptotic Bcl-2 gene *Bax*, and direct binding and inhibition of anti-apoptotic Bcl-2 proteins.

However, combined MDM2 and Bcl-2 inhibition for AML treatment has not been clinically investigated yet. In this study, we sought to confirm the superior activity of this combination and to establish a pre-clinical rationale for its further exploration in clinical studies. We therefore assessed the anti-tumor effects of dual MDM2/Bcl-2 inhibition with the small-molecule inhibitors idasanutlin and venetoclax in p53 wild-type AML models and explored the mechanism of action contributing to their synergistic activity in vitro through cell-cycle analysis, RNA sequencing (RNAseq), and time-course protein expression analysis. Moreover, we confirmed the superior activity of the combination treatment in subcutaneous and orthotopic AML xenograft models.

## Methods

### Reagents

Lyophilized idasanutlin and venetoclax were obtained from Roche Innovation Center Basel and Genentech Inc., South San Francisco, respectively. A stock solution of each was prepared in dimethyl sulfoxide (DMSO), and serial dilutions were prepared in culture medium prior to each experiment; the final DMSO concentration was less than 0.2 % (*v*/*v*) in all experiments.

### Cell lines and culture conditions

The MV4-11 and MOLM-13 p53 wild-type AML cell lines and the p53 mutant HL-60 promyelocytic cell line were obtained from the Roche Pharma Research & Early Development (pRED) cell bank (Roche Diagnostics GmbH). The p53 wild-type/NPM mutant OCI-AML-3 cell line was obtained from the Roche pRED CELLO cell bank (Roche Diagnostics GmbH). MV4-11 and MOLM-13 cells were cultured in RPMI-1640 supplemented with 20 % fetal calf serum (FCS; Gibco), HL-60 cells were cultured in RPMI-1640 supplemented with 10 % FCS, and OCI-AML-3 cells were cultured in alpha-MEM supplemented with 20 % FCS. Cultures were maintained at 37 °C, with 7 % CO_2_ in a humidified atmosphere. Cell line identity was confirmed by short tandem repeats-polymerase chain reaction (STR-PCR) genotyping using the QIAGEN Investigator IDplex Plus Kit (QIAGEN cat. no. 381625).

### Cell viability and apoptosis measurement

Cell cultures were treated with idasanutlin alone (0.6–2000 nM), venetoclax alone (0.6–2000 nM), or both agents combined for 72 h. Viability was measured using CellTiter-Glo® (Promega), as per manufacturer’s instructions, with luminescence measured using the Infinite® F200 microplate reader (Tecan). In short hairpin RNA (shRNA) experiments, viability was measured by trypan blue exclusion using the Cedex HiRes automated analyzer (Roche Diagnostics).

For measurement of apoptosis, cells were stained with Annexin-V-Fluos staining buffer (1 μL/sample Annexin-V-Fluos [Roche Diagnostics GmbH], 2 ng/μL Hoechst 33258, and 400 nM CaCl_2_, prepared in culture medium) and incubated on ice for 15 min in the dark. Fluorescence was analyzed at 4 °C on the LSRII flow cytometer (BD Biosciences).

Data for viability and apoptosis measurements were analyzed using XLfit® for Excel (Microsoft Corporation). Flow cytometric data were analyzed using FlowJo software versions 7.6.5 and 10.0.7 (Treestar).

Relative and absolute half maximal inhibitory concentration (IC_50_) values were calculated for each treatment, with relative IC_50_ corresponding to the mid-point between the upper and lower plateaus of the response curve and absolute IC_50_ representing 50 % of the control.

To determine synergistic effects between idasanutlin and venetoclax, combination indices were calculated as per Loewe and Muchnik [18] using the following equation:

$$ \mathrm{Combination}\ \mathrm{index}=\frac{C_{\mathrm{A}}}{\mathrm{IC}{x}_{\mathrm{A}}}+\frac{C_{\mathrm{B}}}{\mathrm{IC}{x}_{\mathrm{B}}} $$

where IC*x*_A_ and IC*x*_B_ are the concentration of compounds A and B required individually for effect *x* (in this study *x* = relative IC_50_) and C_A_ and C_B_ are the concentration of compounds A and B required in combination to achieve the same effect *x*. Values less than 1 indicate synergy.

### AML xenograft models

Subcutaneous and orthotopic xenograft models were generated in female nude or non-obese diabetic/severe combined immunodeficiency (NOD/SCID) mice. All experiments were conducted by Charles River Discovery Research Services (CR-DRS) in accordance with the recommendations of the Guide for Care and Use of Laboratory Animals [19] with respect to restraint, husbandry, surgical procedures, feed and fluid regulation, and veterinary care. The animal care and use program at CR-DRS is accredited by the Association for Assessment and Accreditation of Laboratory Animal Care International (AAALAC).

For the subcutaneous model, MV4-11 cells (1 × 10^7^) were injected subcutaneously with Matrigel™ Basement Membrane Matrix (BD Biosciences) into the right flanks of 7-week-old mice. On day 12 post-inoculation, mice (*n* = 10 per group) were stratified randomly based on primary tumor size with a median tumor volume (prior to treatment) of approximately 100–150 mm^3^. Idasanutlin (30 mg/kg orally) and venetoclax (100 mg/kg orally) were administered daily for 21 days alone or in combination. Tumor volumes ([length × width]^2^/2) were measured by calipers twice weekly and tumor growth inhibition relative to control animals was calculated as follows:

$$ \mathrm{Tumor}\ \mathrm{growth}\ \mathrm{inhibition} = \left(1-\left[\left(T-T0\right)/\left(C-C0\right)\right]\right) \times 100 $$

where *T* is the tumor volume in the treated group at measurement, *T*0 is the tumor volume in the treated group at baseline, *C* is the tumor volume in the control group at measurement, and *C*0 is the tumor volume in the control group at baseline.

The MV4-11 and MOLM-13 orthotopic models were established by intravenous inoculation of 1 × 10^7^ and 5 × 10^6^ cells, respectively. Ten-week-old mice were pre-treated with cyclophosphamide (150 mg/kg, intraperitoneally), cells were injected into the tail vein, and mice were distributed randomly into four groups (*n* = 10 per group). Treatment was initiated 22 days after inoculation of MV4-11 cells and 3 days after inoculation of MOLM-13 cells. In both tumor studies, idasanutlin (30 mg/kg orally) and venetoclax (100 mg/kg orally) were administered daily for 21 days alone or in combination. Animals were monitored daily for clinical symptoms and adverse events.

A time-to-event (TTE) analysis was conducted until day 78 post-inoculation in the MV4-11 model and day 48 post-inoculation in the MOLM-13 model. The event was death or moribundity due to disseminated leukemia. Any animal that did not reach the endpoint was euthanized at the end of the study and assigned a TTE value of 78 days for the MV4-11 model or 48 days for the MOLM-13 model. Any animal that died from non-tumor burden-related causes was excluded from the analysis. A TTE value was recorded for each assessable animal, and the median TTE was calculated for each group.

The percentage increase in lifespan (ILS) versus the vehicle control was calculated as:

$$ \mathrm{I}\mathrm{L}\mathrm{S}=\frac{\left(T-C\right)}{C} \times 100\% $$

where *T* is the median survival of the treatment group and *C* is the median survival of the control group.

### Cell cycle analysis

MV4-11 and MOLM-13 cells were treated with idasanutlin and venetoclax alone or in combination for 72 h (0.6–2000 nM). At the start of the final 24 h of incubation, 5-bromo-2-deoxyuridine (BrdU; Sigma) was added to cultures at a concentration of 80 μM. Culture medium was also supplemented with 80 μM deoxycytidine (Sigma) at this point to minimize disturbance to the nucleotide pathway. Prior to flow cytometric analysis, cells were washed twice in ice-cold DNA-staining buffer (100 mM Tris pH 7.4, 154 mM NaCl, 1 mM CaCl_2_, 0.5 mM MgCl_2_, 0.1 % NP40, and 0.2 % bovine serum albumin) and incubated in DNA-staining buffer containing 10 U/mL RNase (Roche Diagnostics GmbH) and 1.5 μg/mL Hoechst 33258 for 15 min at 37 °C. Propidium iodide (PI) was added to a final concentration of 1.5 μg/mL, and cells were incubated on ice for 15 min. Fluorescence was analyzed on the LSRII flow cytometer, and data were analyzed using FlowJo software versions 7.6.5 and 10.0.7.

### Gene expression analysis

For mRNA (poly-A) RNAseq studies, MOLM13 cells were treated with idasanutlin (100 nM) and venetoclax (100 nM) alone or in combination for 6 h. High molecular weight RNA (>200 base pairs) was extracted from four biologic replicates using the RNeasy® Mini Kit (QIAGEN®) as per manufacturer’s instructions. Residual genomic DNA was removed during the extraction using the RNase-free DNase set (QIAGEN®). RNA quality was analyzed using Eukaryote Total RNA Nano chips (Agilent Technologies), and all samples used for analysis had an RNA integrity number >8. RNAseq libraries were generated from 1 μg total RNA using the TruSeq® RNA Sample Preparation v2 kit (Illumina®) as per manufacturer’s instructions. Sequencing libraries were quantified using the Kapa Library Quantification kit (Kapa Biosystems), and quality was assessed on the Agilent Bioanalyzer using DNA 1000 chips (Agilent Technologies). Libraries were sequenced on the HiSeq® 2500 sequencer (Illumina) for 2 × 50 cycles using the TruSeq® PE Cluster Kit v3-cBot-HS and TruSeq® SBS Kit v3-HS sequencing reagents (Illumina®). Each lane was spiked with the PhiX Control v3 library (Illumina®) at a final concentration of 1 % (*v*/*v*) as a sequencing control.

Sequencing reads were first aligned to the human protein coding transcriptome (as defined by Ensembl v73 [http://www.ensembl.org]) using Bowtie 2 [20]. In a second step, all reads not mapping to the human transcriptome were aligned to the human genome (hg19) using Bowtie 2. For both mapping steps, default sensitivity settings were used. Gene expression was profiled using in-house tools, and reads per kilobase per million reads (RPKM) were computed as previously described using in-house software [21]. Differential gene expression was computed using DESeq [22]. Genes with an absolute log2 ratio >1 (absolute fold change >2) versus the DMSO control and a false discovery rate corrected *P* value <0.01 were considered differentially expressed. Functional annotation and analysis of altered pathways and functions was performed using Ingenuity® Pathway Analysis (QIAGEN®).

### shRNA analysis

MV4-11 cells (5 × 10^5^) were transduced with MISSION® non-specific or Mcl-1-targeting shRNA lentiviral particles (Sigma) in the presence of polybrene (10 μg/mL); target sequences are listed in Additional file 1. Puromycin (2 μg/mL) was added after 48 h to select for positive transductants, and cells were sampled for viability and protein expression analysis on the following day.

### Western blotting

Cells were lysed in RIPA buffer (Sigma), lysates were loaded onto NuPAGE® 4–12 % Bis-Tris Precast Protein Gels (Life Technologies), and proteins were separated and transferred to nitrocellulose membranes (Life Technologies). Membranes were blocked with 5 % (*w*/*v*) milk powder in Tris-buffered saline with Tween 20 and incubated with Mcl-1, cleaved caspase-3 (Asp175), or ß-actin primary antibodies, followed by incubation with horseradish peroxidase (HRP)-conjugated secondary antibodies (Cell Signaling Technology). Bands were detected using SuperSignal® Chemiluminescent HRP Substrate (Thermo Fisher Scientific).

### Statistical analyses

For viability and apoptosis assays, at least three biologic replicates were analyzed. The data presented are the mean values of one representative experiment. Mean values and standard deviations were calculated using Excel (Microsoft Corporation).

For the MV4-11 subcutaneous model, primary tumor growth data were baseline corrected against tumor volume at the start of treatment, based on ratios. Adjusted data were analyzed using non-parametric methods due to an asymmetric distribution. Treatment-to-control ratios (TCRs) were calculated as follows:

$$ \mathrm{T}\mathrm{C}\mathrm{R} = \frac{{\overline{V}}_{\mathrm{treated}}}{{\overline{V}}_{\mathrm{control}}} $$

where $\overline{V}$ is tumor volume. Two-sided non-parametric confidence intervals (CIs; 1 − *α*) were also calculated [23].

For the MV4-11 and MOLM-13 orthotopic models, the date of the last measurement was used for analysis of median and overall survival and the generation of Kaplan-Meier plots. Treatment groups were compared using a pairwise log-rank test with a multiple test level of 0.00833 considered statistically significant.

Statistical analyses for all in vivo studies were performed using SAS-JMP version 8.1 (SAS Inc., 2007).

## Results

### Combined treatment with idasanutlin and venetoclax shows synergistic effects in AML cell lines in vitro

**Fig. 1:**
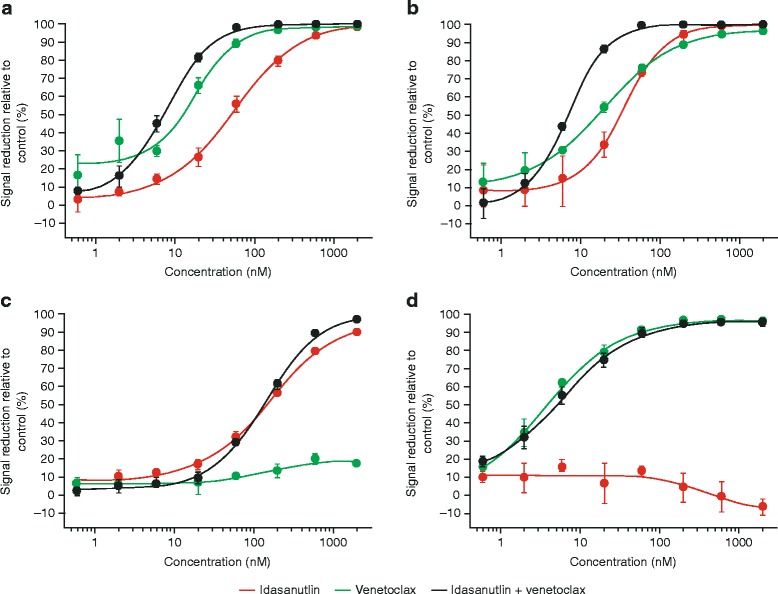
Viability of **a** MV4-11, **b** MOLM-13, **c** OCI-AML-3, and **d** HL60 cells treated with idasanutlin and venetoclax alone and in combination for 72 h. CellTiter-Glo® assay (Promega) was used to determine the cellular viability. Data are displayed as mean plus standard deviation calculated from at least three biological replicates. In MV4-11 cells (**a**), combination treatment led to synergistic effects with a combination index (CI) of 0.72 and a relative IC_50_ of 8 nM compared to 55 and 18 nM for idasanutlin and venetoclax single treatment, respectively. In MOLM-13 cells (**b**), the calculated CI was 0.59 with an IC_50_ of 7 nM for the combination as opposed to single treatment with idasanutlin (IC_50_, 35 nM) and venetoclax (IC_50_, 20 nM). OCI-AML-3 cells were resistant to venetoclax (**c**), while p53 mutated HL-60 cells did not react up to 2 μM idasanutlin as expected (**d**)

The effects of single-agent and combination therapy with idasanutlin and venetoclax were assessed in four AML cell lines exposed to the inhibitors for 72 h. The MV4-11 and MOLM-13 cell lines carry the wild-type *TP53* gene, and the OCI-AML-3 cell line carries wild-type *TP53* and a nucleophosmin (*NPM*) mutation. NPM mutant AML accounts for ~35 % of AML in adults [24]. The HL-60 AML cell line, which carries deletions in both *TP53* alleles, was used as a control to monitor non-specific effects of the MDM2 antagonist idasanutlin. Viability was assessed based on adenosine triphosphate (ATP) concentration, as a measure of metabolically active cells. In viability studies (Fig. 1), dose-dependent effects were seen in the MV4-11 (Fig. 1a) and MOLM-13 (Fig. 1b) p53 wild-type cell lines for single-agent treatment with both inhibitors. Relative and absolute IC_50_ values were calculated for each treatment. For MV4-11 cells, relative IC_50_ values for idasanutlin and venetoclax were 55 and 18 nM, respectively, and absolute IC_50_ values were 51 and 12 nM, respectively. For MOLM-13 cells, the respective relative IC_50_ values were 35 and 20 nM, while absolute IC_50_ values were 31 and 16 nM. In both cell lines, combination treatment was synergistic, with a calculated combination index of 0.72 for MV4-11 cells and 0.59 for MOLM-13 cells (values <1 indicate synergy), corresponding to relative IC_50_ values of 8 and 7 nM, respectively.

Venetoclax had little effect on the viability of the NPM mutant OCI-AML-3 cell line (resistance to Bcl-2 inhibitors has been seen previously in this cell line; data not shown); hence, there was no notable difference between single-agent idasanutlin (relative/absolute IC_50_, 164/147 nM) and the combination treatment for this cell line (relative/absolute IC_50_, 142/133 nM). Non-specific effects were not observed for idasanutlin treatment in the p53 mutant HL-60 line, although venetoclax induced substantial cell death (relative/absolute IC_50_, 4/4 nM).

**Fig. 2:**
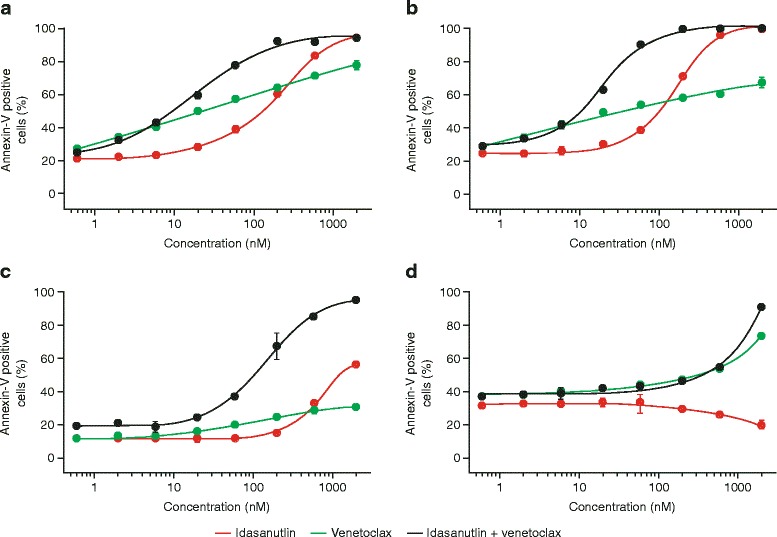
Endpoint apoptosis measurement of **a** MV4-11, **b** MOLM-13, **c** OCI-AML-3, and **d** HL60 cells following treatment with idasanutlin and venetoclax alone and in combination for 72 h. Binding of annexin-V-fluos to phosphatidylserine on the surface of apoptotic cells was assessed using flow cytometry. Data are displayed as mean plus standard deviation calculated from at least three biological replicates. Combination of both compounds led to strongly enhanced cell death induction as compared to single treatment in MV4-11 (**a**) and MOLM-13 (**b**) cells. In OCI-AML-3 cells (**c**), idasanutlin displayed only minor effects, while the combination treatment led to strongly enhanced cell death. Control cell line HL-60 did not respond to idasanutlin treatment (**d**)

The results of cell viability studies were supported by apoptosis analyses using Annexin-V-Hoechst staining (Fig. 2), showing strongly superior effects of the combination treatment for MV4-11 (relative/absolute IC_50_, combination, 19/10 vs. 203/114 nM, idasanutlin single treatment) and MOLM-13 (relative/absolute IC_50_, combination, 20/11 vs. 102/100 nM, idasanutlin single treatment). Again, idasanutlin did not have any effect on p53 mutated HL-60 cells. The OCI-AML-3 cell line did not respond to venetoclax or idasanutlin alone, but was highly sensitive to the combination treatment (relative/absolute IC_50_, 142/108 nM). Given that the viability analyses were based on ATP measurement, the reduction in ATP content relative to the control in this cell line was likely due to G1 cell cycle arrest as opposed to direct cell death, leading to the discrepancy between the viability and apoptosis analyses. Staining of these cells with Hoechst 33258 and PI confirmed G1 cell cycle arrest in idasanutlin- and combination-treated cells. Furthermore, side scatter analysis showed a large proportion of aberrant nuclei with reduced Hoechst 33258 fluorescence, indicating DNA cleavage and confirming that these cells were in the early stages of apoptosis (Additional file 2).

Notably, for venetoclax-induced cell death, the proportion of apoptotic cells measured in the Annexin-V-Hoechst studies did not correspond directly with the results of the viability studies in any of the cell lines analyzed (with the exception of the OCI-AML-3 cell line). This appears to be due to faster apoptosis kinetics in response to venetoclax versus idasanutlin treatment. Given that apoptosis measurement is an endpoint determination, it is possible that Annexin-positive, and even Annexin-Hoechst-positive, cells were missed in this analysis as they had already been degraded.

### Combined treatment with idasanutlin and venetoclax shows superior anti-tumor effects in vivo

**Fig. 3:**
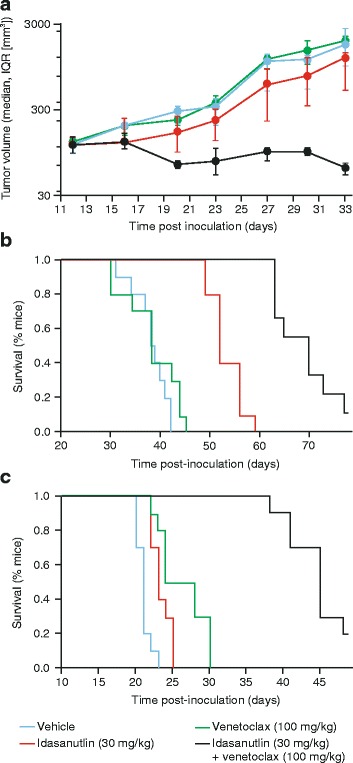
Tumor growth inhibition in **a** MV4-11 subcutaneous model and TTE analysis of survival in **b** MV4-11 and **c** MOLM-13 orthotopic models (*n* = 10 mice per group). Combination treatment resulted in superior anti-tumor activity and enhanced survival. See text for details. *TTE* time-to-event

The activity of idasanutlin and venetoclax were further assessed in subcutaneous and orthotopic AML xenograft models. In the MV4-11 subcutaneous model, tumor growth inhibition relative to the vehicle control group was 0 % for venetoclax (no inhibition vs. control, TCR 0.89, CI, 0.50–2.10) and 30 % for idasanutlin (TCR 0.5, CI, 0.26–0.98). Compared with the single-agent treatments, combination treatment resulted in superior tumor growth inhibition (>100 %) and notably partial tumor regression of 55 % (TCR 0.03, CI, 0.02–0.07) relative to the control (Fig. 3a).

In a TTE analysis of the MV4-11 orthotopic model, the median survival of the mice post-inoculation was 38.5 days for mice treated with the vehicle control (Fig. 3b). In comparison, the median survival was 38 days with venetoclax, 52 days with idasanutlin, and enhanced to 70 days with combination treatment. This corresponded to ILS values of −1 % for venetoclax, 35 % for idasanutlin, and 82 % for combination treatment. The median and overall survival was significantly better for combination treatment than for each of the single-agent treatments (*P* < 0.0001 for both).

In the MOLM-13 orthotopic model, the median survival of the mice post-inoculation was 26 days with venetoclax, 23 days with idasanutlin, and a superior 45 days with combination treatment, compared with 21 days for the vehicle control (Fig. 3c). The corresponding ILS values were 24 % for venetoclax, 10 % for idasanutlin, and 114 % for combination treatment. In terms of efficacy, median and overall survival was significantly better for combination treatment than single-agent treatment (*P* < 0.0001).

For all in vivo studies, treatments were well tolerated as indicated by no significant loss of body weight (>20 %) for the duration of the study.

### Combination treatment leads to accelerated cell death kinetics in vitro

**Fig. 4:**
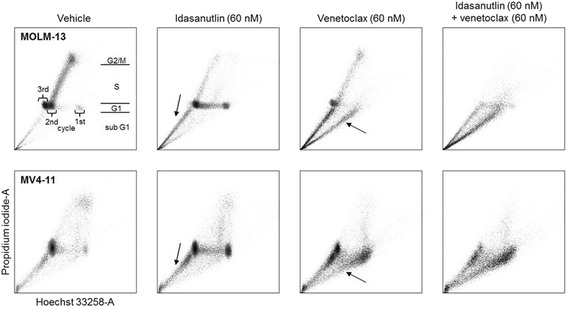
BrdU analysis of cell-cycle kinetics in MOLM-13 and MV4-11 cells treated with idasanutlin and venetoclax alone and in combination for 72 h. BrdU was added for the last 24 h of the experiment as described in the “Methods” section. Populations residing in *G1*, *S*, and *G2/M* phases are indicated as an example for MOLM-13 vehicle-treated cells. *Sub G1* represents nuclear fragmentation as a consequence of cell death. Vehicle (DMSO)-treated cells displayed normal proliferation, with MOLM-13 cells reaching the third and MV4-11 cells reaching the second cycle. Treatment with idasanutlin induces cell cycle arrest and minor nuclear fragmentation from the *G1* phase of the second cell cycle (*sub G1*, *arrows*). Treatment with venetoclax did not affect cell cycle progression, but induced nuclear fragmentation mainly from the *G1* phase of the first cycle (*arrows*). Combination treatment results in remarkably enhanced nuclear fragmentation from the *G1* phase of the first cell cycle onwards with few viable cells remaining

Once the activity of idasanutlin and venetoclax had been assessed in cell culture and human AML xenograft models, exploratory studies were conducted to determine the cellular mechanisms contributing to their combined effects. BrdU analysis using Hoechst 33258 and PI was conducted to analyze changes in cell cycle kinetics of MOLM-13 and MV4-11 cells in response to treatment. BrdU is incorporated into DNA during replication and quenches fluorescence upon subsequent staining with Hoechst, giving a measure of cell division over time. Simultaneous PI staining for DNA content shows cell cycle progression. Figure 4 shows that cells treated with the DMSO control (vehicle) progressed through up to three cell cycles over the 24 h study period as measured by BrdU/Hoechst quenching. Treatment with idasanutlin (60 nM) induced G1 cell cycle arrest in the first and second cycles of replication. Minor nuclear fragmentation indicating apoptosis, evident from a sub-G1 population, was only observed in the G1 phase of the second cycle onwards (Fig. 4). However, treatment with 60 nM venetoclax induced nuclear fragmentation in both the first and second cell cycles (Fig. 4). Combination treatment led to high levels of nuclear fragmentation from cells arrested in the G1 phase of the first cycle and, to a lesser extent, the second cycle (Fig. 4). Hence, viable but non-proliferating cells, arrested in G1 by idasanutlin, are more vulnerable to venetoclax treatment, resulting in enhanced and accelerated cell death kinetics. The observed effects were similar between MV4-11 and MOLM-13 cell lines (Fig. 4).

### Gene expression analysis confirms TP53 pathway activation by idasantulin, while venetoclax treatment does not result in transcriptional changes

RNAseq analysis was performed to measure gene expression changes in response to inhibitor treatment. MOLM-13 cells were exposed to idasanutlin (100 nM) and venetoclax (100 nM) alone or in combination for 6 h, and differential gene expression was analyzed.

**Fig. 5:**
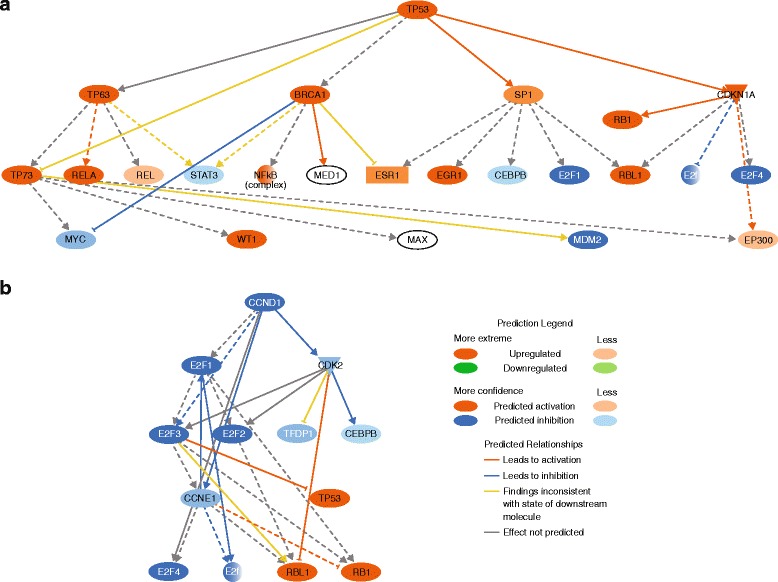
Upstream regulator analysis using Ingenuity pathway software in MOLM-13 cells exposed to idasanutlin (100 nM) for 6 h predicted **a** TP53 pathway activation and **b** CCND1 pathway inhibition. Bcl-2 inhibition by venetoclax did not result in changes of gene transcription

Overall, more than 45 million reads were sequenced for each sample. In comparison with the DMSO control, substantially more genes showed differential expression following idasanutlin and combination treatment than venetoclax treatment. Overall 46, 363, and 183 genes were differentially expressed in response to venetoclax, idasanutlin, and combination treatment, respectively. Upstream regulator analysis using Ingenuity pathway software predicted TP53 pathway activation as expected and CCND1 pathway inhibition indicating G1 arrest as the most significant changes following idasanutlin treatment and also following combination treatment (Fig. 5). No significantly altered pathways were identified in response to venetoclax treatment. There were no obvious differences in gene expression between the idasanutlin-treated cells and combination-treated cells that would explain the synergistic effects of combination treatment in vitro, confirming that venetoclax directly acts on Bcl-2 function rather than on gene expression.

### Combination treatment leads to accelerated Mcl-1 downregulation and caspase-3 activation

**Fig. 6:**
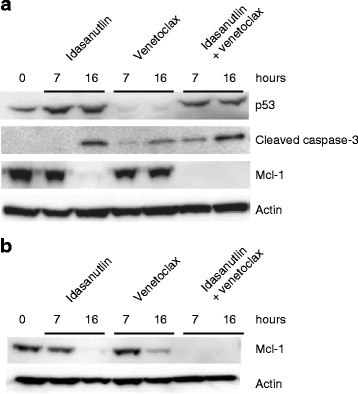
Western blot analysis of **a** p53, cleaved caspase-3, and Mcl-1 protein expression in MOLM-13 cells and **b** Mcl-1 protein expression in MV4-11 cells exposed to venetoclax (100 nM) and idasanutlin (100 nM) alone or in combination for time periods indicated. Actin was used as a loading control for both cell lines

As expected, western blot analysis of MOLM-13 cells showed that p53 protein levels increased with the duration of exposure to idasanutlin and combination treatment, but not with venetoclax treatment (Fig. 6a). Cleaved caspase-3, demonstrating execution of apoptosis, was detected after 16 h of exposure to idasanutlin and after 7 h exposure to venetoclax, although expression levels were lower following venetoclax treatment compared with idasanutlin-treated cells (16 h). Combination treatment with idasanutlin and venetoclax resulted in increased detection of cleaved caspase-3 versus single-agent therapy with either compound from 7 h of exposure. In cells treated with idasanutlin alone, Mcl-1 was still detected after 7 h exposure but was no longer detectable after 16 h, indicating treatment-associated downregulation (Fig. 6a). Single-agent treatment with venetoclax did not inhibit Mcl-1 expression (Fig. 6a). Mcl-1 was not detected at either timepoint following combination treatment, indicating earlier effects on Mcl-1 expression with the combination treatment (Fig. 6a). In MV4-11 cells, Mcl-1 expression was detected at 7 h post-treatment with both inhibitors and at very low levels 16 h post-treatment (Fig. 6b). Consistent with findings in the MOLM-13 cell line, Mcl-1 was not detected in MV4-11 at either timepoint with the combination treatment again indicating accelerated cell death kinetics.

### shRNA knockdown of *Mcl-1* gene expression confirms that Mcl-1 is necessary for AML survival

**Fig. 7:**
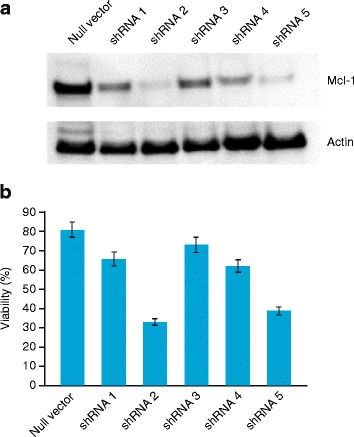
shRNA inhibition of Mcl-1 gene expression in MV4-11 cells **a** Western blot analysis of Mcl-1 protein expression and **b** viability of shRNA transduced cells measured by trypan blue exclusion, confirming that Mcl-1 contributes significantly to AML cell survival

The role of *Mcl-1* in mediating the synergistic effects of idasanutlin and venetoclax in vitro was verified by shRNA experiments in MV4-11 cells (Fig. 7). The MV4-11 cell line was used for this study due to lower expression of Mcl-1 versus MOLM-13 cells (Fig. 6). Transduction of cells with *Mcl-1*-targeting shRNAs leads to *Mcl-1* gene knockdown to varying extents in comparison with the non-specific control (Fig. 7a). This led to decreases in cell viability, with the extent of the decreases corresponding to the extent of Mcl-1 knockdown (Fig. 7b).

## Discussion

Reconstitution of p53 tumor-suppressor activity, through treatment with the MDM2 antagonist idasanutlin, and induction of mitochondrial apoptosis, through specific Bcl-2 inhibition with the BH3 mimetic venetoclax, led to anti-tumor effects in both in vitro and in vivo human xenograft models of AML. The effects of these compounds were significantly enhanced through combination treatment, with combination indices indicating a synergistic effect in vitro. Studies to determine the mechanism(s) underlying this synergy showed that the combination of p53 tumor suppressor signaling and direct induction of mitochondrial apoptosis lead to immediate and potent cell death. Western blot analysis identified that Mcl-1 inhibition is a key contributor to these effects, and earlier Mcl-1 inhibition is seen in response to idasanutlin/venetoclax combination treatment, correlating with accelerated cell death kinetics.

Both nutlins and BH3 mimetics have shown promise in pre-clinical and clinical studies [5, 6, 25–29]. Idasanutlin and venetoclax represent clinically optimized molecules within their respective compound classes. Idasanutlin shows increased specificity and activity, with lower dosage requirements, compared with preceding nutlin-class compounds [5, 8]. Venetoclax has enhanced specificity compared with earlier BH3 mimetics (such as ABT-737 and the related clinical-grade compound navitoclax), which interact with the BH3 domains of Bcl-2, Bcl-xL, and Bcl-w. Venetoclax specifically inhibits Bcl-2 and therefore is not associated with the dose-limiting thrombocytopenia resulting from Bcl-xL inhibition [30].

Our data show that exposure of human AML cell lines in vitro to single-agent venetoclax or idasanutlin induced cell death without off-target effects, with enhanced apoptotic activity seen when cells were treated with the two agents in combination. Discrepancies in the levels of inhibition between the cell viability and apoptosis studies are likely due to the nature of the assays used and highlight the different modes of action of the two inhibitors. The viability studies (Fig. 1) were based on ATP measurement as an indicator of metabolic activity, while apoptosis studies (Fig. 2) measured phosphatidyl serine exposure and membrane permeability. Thus, the viability assays will detect cell cycle arrest in addition to apoptosis. For idasanutlin, cells became arrested in the G1 phase of the cell cycle initially before induction of apoptosis in subsequent cycles (Fig. 4). This is illustrated in the OCI-AML-3 cell line where there was no difference observed between idasanutlin and combination treatment in viability studies based on ATP concentration. However, in apoptosis studies, there was little cell death in response to idasanutlin, indicating that the effects seen in the viability studies were due to cell cycle arrest as opposed to cell death (Figs. 1 and 2 and Additional file 2). In the MV4-11 and MOLM-13 cell lines, induction of cell death was initiated immediately in response to venetoclax treatment and this may actually have led to some apoptotic cells being missed by the assay. This difference in cell death kinetics between the two inhibitors was confirmed by Western blotting analysis of cleaved caspase-3 expression, which shows accelerated induction of apoptosis in response to venetoclax versus idasanutlin (Fig. 6).

Notably, for the OCI-AML-3 cell line, little inhibitory effect was seen in response to venetoclax treatment and this may be due to BH3 mimetic resistance previously observed with this cell line (data not shown). However, combination treatment induced substantial cell death in this cell line, overcoming resistance to venetoclax and removing the cell cycle dependency of idasanutlin activity (Figs. 1 and 2 and Additional file 2).

In contrast to the in vitro studies, more modest growth inhibition was seen when the single-agent inhibitors were administered to in vivo human AML xenograft models. Venetoclax had no effect in either the subcutaneous or orthotopic MV4-11 models and neither inhibitor showed substantial improvements in survival versus the vehicle control in the MOLM-13 model, although venetoclax was marginally better than idasanutlin. This low activity for single-agent treatment may be due to the aggressive nature of these in vivo AML models, particularly the MOLM-13 orthotopic model, or due to poor tumor penetration of the compounds. In spite of this and in line with our results in vitro, combination treatment led to superior tumor growth inhibition and substantial ILS compared with the vehicle control and single-agent therapies.

Our data confirm and add on to previously published findings. Saiki et al. [31] showed synergistic effects when combining MDM2 antagonists and the dual Bcl-2/Bcl-xL inhibitors ABT-737 and ABT-263 in solid and hematologic tumor cell lines. Using the Bcl-2-selective inhibitor ABT-199 as combination partner, activity was mainly restricted to hematological cell lines. The authors concluded that Bcl-xL inhibition is essential for synergy with MDM2 antagonists in solid tumor cell lines, while selective Bcl-2 inhibition in hematologic cell lines is sufficient [31]. Moreover, an earlier study of dual MDM2 and Bcl-2 inhibition with ABT-737 and nutlin-3 (tool compounds used for in vitro studies) showed the feasibility of combining MDM2 and Bcl-2 inhibitors in AML [32]. In that study, the individual effects of MDM2 and Bcl-2 inhibition were shown to be cell cycle-specific with nutlin-3a inducing apoptosis predominantly in the G2/M phase of the cell cycle while ABT-737 induced apoptosis predominantly while cells were in G1. Combination treatment removed the cell cycle-dependent effects of treatment, with the authors proposing that this could account, in part, for the synergy observed. Similar cell cycle effects were seen in the current study in response to single-agent exposure to the clinical-grade compounds idasanutlin and venetoclax. Treatment with idasanutlin alone led to G1 cell cycle arrest, with little evidence of apoptosis until subsequent cycles of replication. With venetoclax treatment, cells continued to divide and apoptosis was induced during each round of replication. Combination treatment resulted in direct induction of cell death without any further cell division. Thus, the cell cycle-independent activity of the combination treatment could contribute to the synergy, which manifests as an acceleration of cell death kinetics.

Gene expression analysis in the MOLM-13 cell line further highlighted the different mechanisms of action of venetoclax and idasanutlin. The expression of far fewer genes was altered in response to venetoclax treatment compared with idasanutlin or combination treatment, reflecting the fact that Bcl-2 inhibition and subsequent mitochondrial apoptosis is mediated mainly through protein-protein interactions while p53 signaling is mediated at the protein and transcriptional level. Upstream regulator analysis of gene expression changes in response to idasanutlin and combination treatment identified TP53 pathway activation and CCND1 pathway inhibition as being significant (Fig. 5), which is as expected based on the cell cycle inhibition observed in the cell cycle kinetic experiments (Fig. 4).

The concept that Mcl-1 might be an important mediator of AML cell survival has been described in previous work [33]. By shRNA and Western blot studies, we confirmed that Mcl-1 inhibition appears to play a significant role in maintaining viability of AML cells (Figs. 6 and 7). Moreover, we demonstrated for the first time that the synergistic effects seen in vitro in combination treatment with venetoclax and idasanutlin is due to accelerated Mcl-1 downregulation and subsequent induction of apoptosis (Fig. 6). This is notable, as Mcl-1 expression is also associated with resistance to BH3 mimetics [34]. Suppression of Mcl-1 expression here in response to idasanutlin treatment may increase the sensitivity of AML cells to venetoclax. Mcl-1 degradation through activated caspases at the timepoints investigated is rather unlikely, since Mcl-1 in MOLM-13 cells was still detectable after cleaved-caspase-3 was already induced (Fig. 6a). Our findings are consistent with a previous study that showed decreased expression of Mcl-1 in response to cyclin-dependent kinase inhibition which led to increased sensitivity of human leukemia cells to the BH3 mimetic ABT-737 [35].

## Conclusions

In this study, we provided evidence that in human AML models, venetoclax and idasanutlin display a strongly superior effect when administered in combination to provide substantial improvements in anti-tumor activity versus the respective single-agent treatments. Combination treatment removed the cell cycle dependency of idasanutlin response leading to an acceleration of cell death kinetics versus single-agent treatment. It also led to enhanced inhibition of the anti-apoptotic protein Mcl-1, a known resistance factor to BH3 mimetics including venetoclax. Given the clinical efficacy that has already been shown for idasanutlin and venetoclax as single agents in AML [29, 30], these findings support the further testing of this novel combination in clinical studies. In addition, pre-selecting patient populations on the basis of p53 status and Bcl-2 expression might significantly accelerate clinical development of this combination [36]. To this end, a clinical study with idasanutlin and venetoclax is being initiated currently.

## Abbreviations

AML, acute lymphoblastic leukemiaBH3, BCL-2 homolog domain 3; BrdU, 5-bromo-2-deoxyuridine; CCND1, gene encoding cyclin-D1; CI, confidence interval; CLL, chronic lymphatic leukemia; DMSO, dimethyl sulfoxide; FCS, fetal calf serum; FDA, United States Food and Drug Administration; HRP, horseradish peroxidase; IC_50_, half maximal inhibitory concentration; ILS, increase in lifespan; MDM2, mouse double minute 2 homolog; NOD/SCID, non-obese diabetic/severe combined immunodeficiency; PCR, polymerase chain reaction; PI, propidium iodide; RNAseq, RNA sequencing; shRNA, short hairpin RNA; STR, short tandem repeats; TCR, treatment-to-control-ratio; TTE, time-to-event

## Additional files

Additional file 1: Vector sequences for Mcl-1-targeting shRNA. (DOCX 13 kb) Additional file 2: Flow cytometric analysis of OCI-AML-3 cells following exposure to venetoclax and idasanutlin alone or in combination for 72 h. BrdU was added for the last 24 h as described in the “Methods” section. The top panel shows PI fluorescence versus SSC-A with gating for normal nuclei, abberant (SSC-A high) nuclei and subG1 events. The bottom panel shows cell cycle distribution for these subsets stained with Hoechst 33258 and PI. As expected, idasanutlin induced cell cycle arrest in G1, first cycle, while venetoclax had little or no effect on cell cycle progression and viability. The combination of both compounds lead to an increase of SSC-A high events. Events in subG1 were mainly derived from aberrant nuclei, while some SSC-A high nuclei are still intact. The decrease in Hoechst 33258 fluorescence indicated that DNA degradation has already started, confirming that these cells were in the early stages of apoptosis. PI, propidium iodide; SSC-A, side scatter area. (PDF 73 kb)
