# Supplementary material for: Superior anti-tumor activity of the MDM2 antagonist idasanutlin and the Bcl-2 inhibitor venetoclax in p53 wild-type acute myeloid leukemia models
Source: J Hematol Oncol. 2016 Jun 28;9:50. doi: 10.1186/s13045-016-0280-3 (PMC4924270; doi:10.1186/s13045-016-0280-3)
Supplement: Additional file 1: — Vector sequences for Mcl-1-targeting shRNA. (DOCX 13 kb) [file 13045_2016_280_MOESM1_ESM.docx]

**Additional Material**

**Additional File 1**

**Vector Sequences for Mcl-1-Targeting shRNA:**

TRCN0000194663: CCGGCTGATAACTATGCAGGTTTAACTCGAGTTAAACCTGCATAGT

TATCAGTTTTTTG

TRCN0000005514: CCGGGCTGTGTTAAACCTCAGAGTTCTCGAGAACTCTGAGGTTTAA

CACAGCTTTTT

TRCN0000005516: CCGGGCTGGAGATTATCTCTCGGTACTCGAGTACCGAGAGATAAT

CTCCAGCTTTTT

TRCN0000005517: CCGGGCTAAACACTTGAAGACCATACTCGAGTATGGTCTTCAAGTG

TTTAGCTTTTT

TRCN0000005518: CCGGGCTTCGGAAACTGGACATCAACTCGAGTTGATGTCCAGTTTC

CGAAGCTTTTT
