# Supplementary figures and images for: Superior anti-tumor activity of the MDM2 antagonist idasanutlin and the Bcl-2 inhibitor venetoclax in p53 wild-type acute myeloid leukemia models
Source: J Hematol Oncol. 2016 Jun 28;9:50. doi: 10.1186/s13045-016-0280-3 (PMC4924270; doi:10.1186/s13045-016-0280-3)

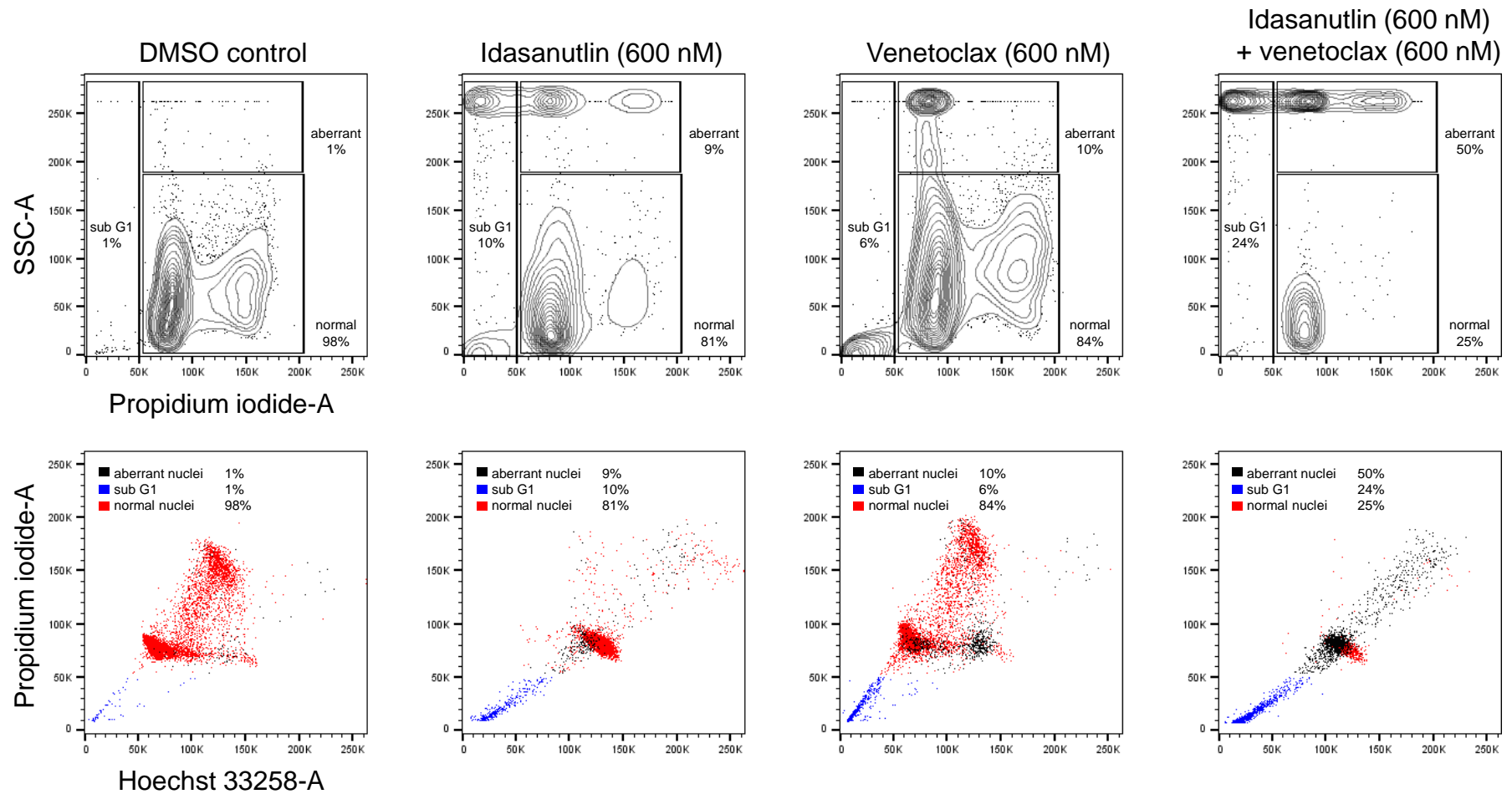

Supplement: Additional file 2: — Flow cytometric analysis of OCI-AML-3 cells following exposure to venetoclax and idasanutlin alone or in combination for 72 h. BrdU was added for the last 24 h as described in the “Methods” section. The top panel shows PI fluorescence versus SSC-A with gating for normal nuclei, abberant (SSC-A high) nuclei and subG1 events. The bottom panel shows cell cycle distribution for these subsets stained with Hoechst 33258 and PI. As expected, idasanutlin induced cell cycle arrest in G1, first cycle, while venetoclax had little or no effect on cell cycle progression and viability. The combination of both compounds lead to an increase of SSC-A high events. Events in subG1 were mainly derived from aberrant nuclei, while some SSC-A high nuclei are still intact. The decrease in Hoechst 33258 fluorescence indicated that DNA degradation has already started, confirming that these cells were in the early stages of apoptosis. PI, propidium iodide; SSC-A, side scatter area. (PDF 73 kb) [file 13045_2016_280_MOESM2_ESM.pdf]
